# Retraction: AKAP13 Rho-GEF and PKD-Binding Domain Deficient Mice Develop Normally but Have an Abnormal Response to β-Adrenergic-Induced Cardiac Hypertrophy

**DOI:** 10.1371/journal.pone.0296004

**Published:** 2023-12-12

**Authors:** The PLOS ONE Editors

Following the publication of this article [1], concerns were raised regarding Fig 2.

Specifically:

- In Fig 2B, AKAP LBC V5 panel band 1 appears similar to band 4 when horizontally flipped.
- In Fig 2D:
  ○ Bands 1, 2, and 4 of the AKAP Lbc V5 panel appear similar when band 2 is horizontally flipped. Additionally, these bands appear similar to band 1 in the AKAP Lbc V5 panel in Fig 2C.
  ○ When levels are adjusted to visualize the background, there appear to be vertical discontinuities in the AKAP Lbc V5 panel between lanes 1 and 2 and lanes 3 and 4.

The authors stated that the underlying data for the figures of concern could not be recovered, as the Carnegie lab, which conducted these experiments, closed following his death in 2014. Regarding the concern in Fig 2B, the authors provided a raw blot from a replicate experiment corroborating the data provided in the published panel. The authors additionally provided individual level data for the RHO-Gef and PKA activity assays in Fig 2, but were unable to provide the original underlying data for the blots in Fig 2C and 2D. The PLOS Editors remain concerned about the unresolved issues in these panels.

In light of the concerns affecting multiple panels in Fig 2 that question the reliability of these data, the *PLOS ONE* Editors retract this article.

The corresponding author noted that GC is deceased. MJSpindler, BTB, ECH, NS, DS, and BRC agreed with the retraction. YH and MJScott either did not respond directly or could not be reached.
